# Colonic Perforation in a Child with Crohn's Disease: Successful Medical Treatment Rescues from Colectomy

**DOI:** 10.1155/2012/152414

**Published:** 2012-09-29

**Authors:** Marco Gasparetto, Benedetta Giorgi, Wolfgang Kleon, Faise Al Bunni, Graziella Guariso

**Affiliations:** ^1^Department of Paediatrics, Unit of Gastroenterology, Digestive Endoscopy, Hepatology, and Care of the Child with Liver Transplantation, University Hospital of Padova, Via Giustiniani 3, 35128 Padova, Italy; ^2^Department of Medical Diagnostic Sciences and Special Therapies, Institute of Radiology, University Hospital of Padova, Via Giustiniani 1, 35128 Padova, Italy; ^3^Unit of Paediatrics, Central Hospital of Bolzano, Via Lorenz Böhler 5, 39100 Bolzano, Italy

## Abstract

*Background*. The challenging treatment of penetrating paediatric Crohn's disease (CD) involves pharmacological and surgical approaches. Despite a proved efficacy of anti-TNF agents for treatment of complex fistula, a large number of patients cannot achieve a complete healing and relapse during the followup. *Aim*. We report a paediatric case with CD and colonic perforation who was successfully treated with medical therapy only, including anti-TNF**α**. *Case Presentation*. During a colonoscopy performed on a 9-year-old girl with CD, a perforation occurred in correspondence of a fistula at the colonic splenic flexure. The formation of a collection was then detected (US, enteric-CT), as well as a fistula connecting the colon to the collection. The girl was kept fasting and treated with total parenteral nutrition and antibiotic therapy. Treatment with Infliximab was also started, and after the third dose a US control showed disappearance of the collection and healing of the enteric fistula. Parenteral nutrition was progressively substituted with enteral feeding, and no surgical treatments were needed. *Discussion*. In pubertal children with penetrating CD, the option of an efficacious medical treatment to avoid a major surgical approach on the bowel is to be aimed for growth improvement. This approach requires a strictly monitored long-term followup.

## 1. Introduction

Many recent studies on therapeutic availabilities for inflammatory bowel disease (IBD) have evidenced that a top-down treatment for the more aggressive phenotypes of disease relates to a better outcome and is associated with a prolonged remission within the long-term followup. This approach can reduce the need of multiple further treatments. It is crucial to identify those patients who deserve a top-down approach in order to sustain their growth and pubertal development until and throughout adolescence [1–3].

A surgical intervention is generally to be considered as a last option for IBD; nevertheless it happens to be required in 50–70% of cases with Crohn's disease (CD) within 10–15 years since diagnosis [4–6].

The inefficacy of medical treatment in controlling the activity of disease in IBD patients with unacceptable quality of life is the most common indication to a surgical intervention [7, 8].

We here report the case of a young girl with penetrating CD and formation of a perisplenic and retrocolonic collection, who responded successfully to a medical treatment including anti-TNF-alphas and was able to avoid surgical intervention, with a subsequent brilliant recuperation in linear weight and pubertal growth as well as in quality of life.

## 2. Case Presentation

The clinical history of a caucasian 9-year-old girl began with the appearance of diffuse abdominal pain, fatigue, anorexia, and progressive weight loss.

As these symptoms had been worsening throughout the following six months, a diagnostic upper and lower endoscopy was performed: multiple ulcers, adherent fibrin, and pseudopolyps were observed at the transverse colon and left colon. The Bauhin valve was stenotic. Superficial ulcers and erosions were seen also at the terminal ileum. The histological exam confirmed a pattern being compatible with CD. 

The endoscopic procedure was complicated by a perforation at the level of the splenic colonic flexure, which required an urgent laparoscopic treatment with suture of the colonic wall. During the subsequent followup, the formation of a perisplenic collection was detected at ultrasound (US) and CT, with concomitant increase in the pancreatic enzymes. 

After an initial improvement in the clinical general conditions with parenteral antibiotic treatment, a severe abdominal pain reappeared with main localisation on the left side, together with fever, leucocytosis, and increase in inflammation indexes (CRP 120 mg/L). An intensification of the immunosuppressive treatment was therefore performed adding Azathioprine to the ongoing corticosteroid therapy. 

**Figure 1:**
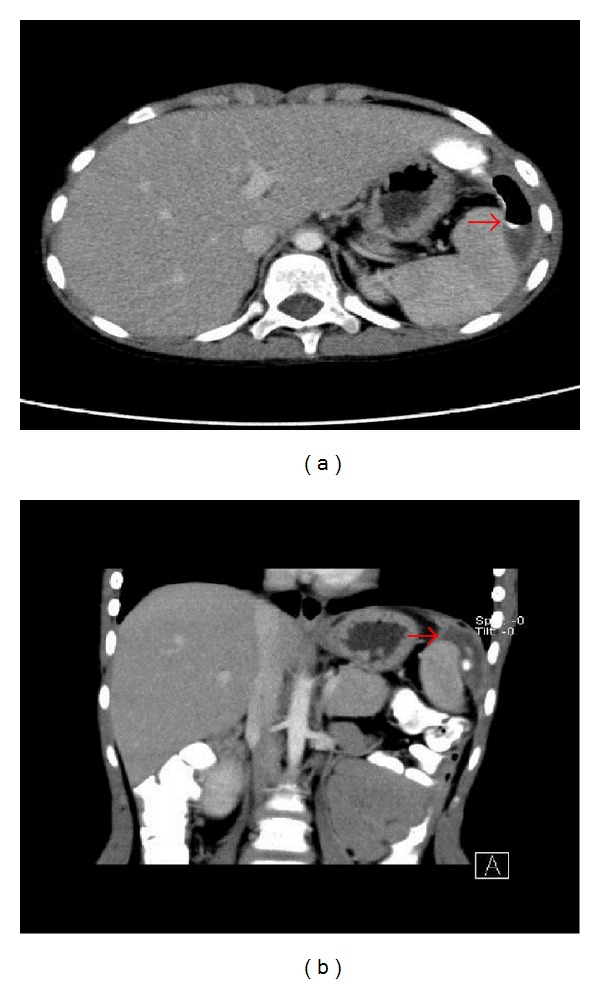
Axial (a) and Coronal (b) abdominal CT projections (with enteric contrast) demonstrate a perisplenic hydroaerial collection with a dense component compatible with enteric content (arrows). A thin fistula (*) connects the collection to the colonic lumen. A further retroperitoneal fluid collection is detectable at the left parietal-colonic region.

An abdominal CT performed (Figure 1) evidenced a perisplenic hydroaerial collection with a dense component which was compatible with enteric content; a thin fistula connecting the collection to the colonic lumen was also depicted, as well as a further retroperitoneal fluid collection at the left parietal-colonic region.

Ten days later, the child was transferred to our Unit of Paediatric Gastroenterology appearing febrile, undernourished, and in compromised conditions. 

The anatomical characteristics of the collection and the diagnosis of active CD determined major surgical concerns for a selective drainage. A surgical alternative proposed was total colectomy with ileostomy. 

We decided instead to choose a medical treatment which initially consisted in long-term parenteral antibiotic therapy, parenteral nutrition, and complete fasting. Azathioprine and corticosteroid treatment were suspended.

**Figure 2:**
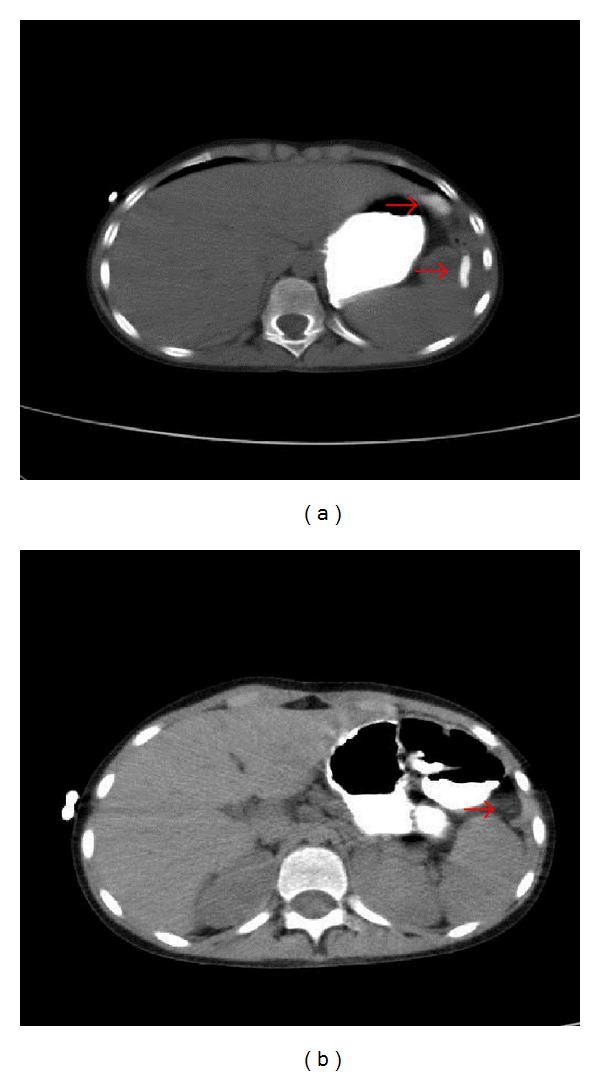
Axial abdominal CT projections (with enteric contrast enhancement) showing a progressive healing of the fistula connecting the colonic lumen to the collection before (a) and after (b) the second administration of anti-TNF-*α*.  Figure 2(a). detects the passage of enteric contrast from the colon into the parietal-colonic collection, which is no more visible in Figure 2(b).

A close monitoring of the collection and of the fistula was performed through CT (Figure 2) and US images. 

The child progressively recovered better clinical conditions with disappearance of fever, abdominal pain, and decrease in inflammation indexes and pancreatic enzymes.

A first attempt with enteral nutrition evidenced an increase in the dimensions of the abdominal mass at a US control, due to the passage of the intestinal content through the fistula until the reaching of the perisplenic and retroperitoneal region where the previous collection was still located.

A complete fasting was thus indicated.

A progressive improvement in general condition and nutritional status, together with a gradual reduction in the dimensions of the abdominal collection until the disappearance of its parietal-colonic component was observed. 

After one month of treatment, keeping a complete fasting and the parenteral antibiotic therapy, a minimal perisplenic collection and a thin colonic fistula were still detectable; a first dose of anti-TNF-alpha (Infliximab 5 mg/Kg) was administered attesting good toleration without any adverse effect. The subsequent radiologic controls evidenced a major reduction in the dimensions of the perisplenic collection, (2 × 1.5 cm) and the absence of any passage of the oral mean of contrast (Gastrographin) through the fistula. 

At this point, after one month and a half since the admission at our Unit, the child was allowed to introduce orally a small amount of exclusive polymeric formula and liquids. No complications were attested until the administration of the second dose of Infliximab (2 weeks after the first dose).

The subsequent radiological controls evidenced a complete disappearance of the fistula as well as of the perisplenic collection.

The child did not present any clinical symptom. Her nutritional condition at discharge was significantly improved with a gain of 4 kilograms in body weight in 2 months. The girl is now proceeding with anti-TNF-alpha treatment and has completely suspended parenteral nutrition since one month and a half after discharge. Her height and body weight are now both corresponding to the 50^∧^ percentile for age. The child refers an excellent quality of life.

## 3. Discussion

The anti-TNF-*α* Infliximab is at present the only proved drug to be able to modify the natural history of IBD [1, 3, 4, 9, 10].

Infliximab efficacy for induction and maintenance of remission in children and adults with severe penetrating Crohn's disease who are nonresponders to the conventional therapy, is observed in terms of mucosal healing, decrease in incidence of complications and hospitalisations, improvement in extraintestinal manifestations and, above all, in the process of child growth [1, 3, 9]. The efficacy of Infliximab is usually more observed within the paediatric population than among adult patients, as regards both the induction and maintenance of remission. Nevertheless, any adverse effect of anti-TNF-*α* treatment is less tolerated among children who present more problems related to adverse effects and reduced compliance [11].

It is evidenced that in children and adolescents with growth delay, treatment with Infliximab reestablishes the normal linear growth rate and can also recuperate the pubertal development. 

The presence of nonperianal fistula (i.e., enteric-enteric, enteric-cutaneous, and enteral-urinary) significantly reduces the response to biological treatment (which decreases to about 20%), probably because for these lesions the mechanical component predominates on the inflammatory component; the surgical solution can therefore be the unique efficacious alternative for these cases [4, 5]. 

A surgical intervention represents a psychologically important step to be faced by young patients and their families; an appropriate counselling and a psychological preparation is determinant to increase patients' copying [4].

The morbidity related to active IBD and its progression needs to be balanced against the morbidity of a surgical intervention. 

Crohn's disease potentially involves the whole gastrointestinal tract, so a surgical intervention does not represent a curative solution since the disease may relapse in any other remaining part of the bowel. The risk for relapse is generally higher for the perforating phenotype than for the stenosing one. One year after surgery, 75% of patients presents new lesions at the endoscopic evaluation whereas a 40–53% presents relapses for which a new surgical intervention is needed [1].

What arises from this clinical experience are the potential benefits of a medical approach (including anti-TNF alpha) in cases of paediatric complicated penetrating CD. In pubertal children with IBD, the option of an efficacious medical treatment with avoidance or postposition of a major surgical approach on the bowel (i.e., colectomy, ileostomy) is to be aimed for growth improvement. 

This approach requires a strictly monitored long-term followup. 

We consider this case experience a significant proof of the potentialities of a well-conducted medical therapy for those severe cases of IBD with an unfavourable risk-benefit balance for surgical options.

## Abbreviations

CD: Crohn's disease
CRP: C reactive protein
CT: Computerized tomography
ESR: Erythrocytes sedimentation rate
IBD: Inflammatory bowel disease
US: Ultrasound
TNF*α*: Tumor necrosis factor alpha.
